# HbA_1c_ Measured in Stored Erythrocytes Is Positively Linearly Associated with Mortality in Individuals with Diabetes Mellitus

**DOI:** 10.1371/journal.pone.0038877

**Published:** 2012-06-13

**Authors:** Diewertje Sluik, Heiner Boeing, Jukka Montonen, Rudolf Kaaks, Annekatrin Lukanova, Annelli Sandbaek, Kim Overvad, Larraitz Arriola, Eva Ardanaz, Calogero Saieva, Sara Grioni, Rosario Tumino, Carlotta Sacerdote, Amalia Mattiello, Annemieke M. W. Spijkerman, Daphne L. van der A, Joline W. J. Beulens, Susan van Dieren, Peter M. Nilsson, Leif C. Groop, Paul W. Franks, Olov Rolandsson, Bas Bueno-de-Mesquita, Ute Nöthlings

**Affiliations:** 1 Department of Epidemiology, German Institute of Human Nutrition Potsdam-Rehbruecke, Nuthetal, Germany; 2 Division of Cancer Epidemiology, German Cancer Research Centre, Heidelberg, Germany; 3 Department of General Practice, School of Public Health, Aarhus University, Aarhus, Denmark; 4 Department of Epidemiology, School of Public Health, Aarhus University, Aarhus, Denmark; 5 Public Health Department of Gipuzkoa, Basque Government, San Sebastián, Spain; 6 Consortium for Biomedical Research in Epidemiology and Public Health, San Sebastián, Spain; 7 Consortium for Biomedical Research in Epidemiology and Public Health, Pamplona, Spain; 8 Navarre Public Health Institute, Pamplona, Spain; 9 Molecular and Nutritional Epidemiology Unit, Cancer Research and Prevention Institute, Florence, Italy; 10 Nutritional Epidemiology Unit, National Cancer Institute, Milan, Italy; 11 Cancer Registry and Histopathology Unit, “Civile – M.P. Arezzo” Hospital, Ragusa, Italy; 12 Piedmont Centre for Cancer Prevention, Turin, Italy; 13 Human Genetic Foundation, Turin, Italy; 14 Department of Clinical and Experimental Medicine, Federico II University, Naples, Italy; 15 Center for Prevention and Health Services Research, National Institute for Public Health and the Environment, Bilthoven, The Netherlands; 16 National Institute for Public Health and the Environment, Bilthoven, the Netherlands; 17 Julius Centre for Health Sciences and Primary Care, University Medical Centre Utrecht, Utrecht, The Netherlands; 18 Department of Clinical Sciences, Lund University, Malmö, Sweden; 19 Divisions of Medicine and Nutritional Research, Department of Public Health and Clinical Medicine, Umeå University, Umeå, Sweden; 20 Genetic and Molecular Epidemiology Unit, Department of Clinical Sciences, Skåne University Hospital, Lund University, Malmö, Sweden; 21 Department of Nutrition, Harvard School of Public Health, Boston, Massachusetts, United States of America; 22 Family Medicine, Department of Public Health and Clinical Medicine, Umeå University, Umeå, Sweden; 23 Department of Gastroenterology and Hepatology, University Medical Centre Utrecht, Utrecht, The Netherlands; 24 Epidemiology Section, Institute for Experimental Medicine, Christian-Albrechts-University of Kiel, Kiel, Germany; Tehran University of Medical Sciences, Islamic Republic of Iran

## Abstract

**Introduction:**

Observational studies have shown that glycated haemoglobin (HbA_1c_) is related to mortality, but the shape of the association is less clear. Furthermore, disease duration and medication may modify this association. This observational study explored the association between HbA_1c_ measured in stored erythrocytes and mortality. Secondly, it was assessed whether disease duration and medication use influenced the estimates or were independently associated with mortality.

**Methods:**

Within the European Prospective Investigation into Cancer and Nutrition a cohort was analysed of 4,345 individuals with a confirmed diagnosis of diabetes at enrolment. HbA_1c_ was measured in blood samples stored up to 19 years. Multivariable Cox proportional hazard regression models for all-cause mortality investigated HbA_1c_ in quartiles as well as per 1% increment, diabetes medication in seven categories of insulin and oral hypoglycaemic agents, and disease duration in quartiles.

**Results:**

After a median follow-up of 9.3 years, 460 participants died. Higher HbA_1c_ was associated with higher mortality: Hazard Ratio for 1%-increase was 1.11 (95% CI 1.06, 1.17). This association was linear (P-nonlinearity =0.15) and persistent across categories of medication use, disease duration, and co-morbidities. Compared with metformin, other medication types were not associated with mortality. Longer disease duration was associated with mortality, but not after adjustment for HbA_1c_ and medication.

**Conclusion:**

This prospective study showed that persons with lower HbA_1c_ had better survival than those with higher HbA_1c_. The association was linear and independent of disease duration, type of medication use, and presence of co-morbidities. Any improvement of HbA_1c_ appears to be associated with reduced mortality risk.

## Introduction

Glycemic control is the main objective of diabetes management to reduce risk of complications. Glycated hemoglobin (HbA_1c_) provides a measure of average blood glucose levels over the preceding two to three months and is considered the best measurement of long-term glycemic control [1]. The American Diabetes Association (ADA) advises diabetes patients to achieve and maintain an HbA_1c_ below 7% [1].

**Figure 1:**
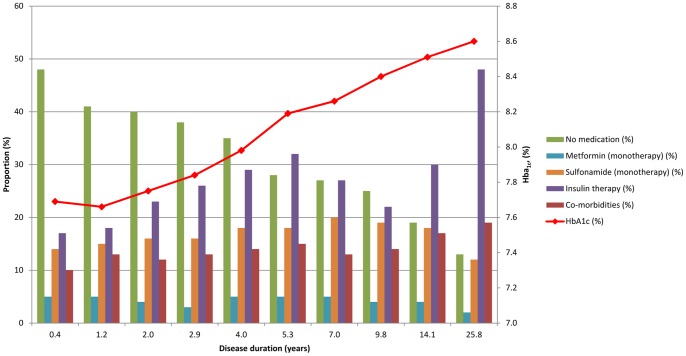
Proportions of Medication Use and Co-Morbidities, and HbA_1c_ from Stored Erythrocytes across Deciles of Disease Duration (years) in 5,837 Individuals with Diabetes.

The United Kingdom Prospective Diabetes Study Group (UKPDS) has shown that intensive blood-glucose control reduced microvascular complications [2] and improvement in glycemic control across all ranges of achieved HbA_1c_ values, was associated with a reduced risk of diabetes complications [3]. A meta-analysis of five randomized controlled trials showed that intensive glycemic control reduced coronary events but was not associated with overall mortality compared with standard control [4]. Furthermore, several observational studies showed that elevations in HbA_1c_ were associated with ischemic heart disease mortality [5], cardiovascular disease (CVD) [6]–[8], and total mortality [6], [8]. Other studies, however, have suggested that the association between HbA_1c_ and mortality might be U-shaped: Currie *et* *al.* reported a higher mortality risk in diabetics with observed HbA_1c_ lower than 7.5% [9] and the Action to Control Cardiovascular Risk in Diabetes (ACCORD) Study Group suggest that intensive glycemic control for 3.7 years to target a HbA_1c_ below 6% increases 5-year mortality from any cause in a group of individuals with type 2 diabetes [10]. However, this higher mortality risk associated with lower achieved HbA_1c_ levels could have been due to other factors: age, disease duration and presence of co-morbidities should to be taken into account [11]–[13].

**Table 1:** Baseline Characteristics^a^ of 4,345 Individuals with Diabetes from the European Prospective Investigation into Cancer and Nutrition across Study Center-Specific Quartiles of HbA_1c_ Measured in Stored Erythrocytes.

	Q1		Q2		Q3		Q4	
	Mean (SE)	%	Mean (SE)	%	Mean (SE)	%	Mean (SE)	%
N	1,162		1,054		1,071		1,058	
HbA_1c_, %	6.3 (0.5)		7.3 (0.6)		8.3 (0.9)		10.6 (1.8)	
Male participants		51		54		52		51
Age, y	56.4 (7.7)		57.4 (7.0)		57.8 (7.0)		57.3 (6.9)	
Disease duration, y	7.0 (7.5)		7.7 (7.9)		9.3 (8.5)		10.5 (8.5)	
No medication use		51		37		22		14
Metformin (monotherapy)		5		7		6		4
Metformin (combined)		8		11		13		15
Sulfonamides		17		19		22		23
Other OHA or combinations		5		7		7		7
Insulin and OHA combined		3		5		8		10
Insulin therapy		11		16		23		27
Systolic blood pressure, mmHg	141 (20)		144 (21)		144 (20)		145 (20)	
Co-morbidities		10		10		12		12
BMI, kg/m^2^	28.4 (4.6)		28.9 (4.8)		29.0 (4.8)		29.4 (4.9)	
Smoking status								
Never		42		43		40		39
Former		34		34		35		36
Current		23		22		24		24
Physical activity								
Inactive		14		14		13		11
Moderately inactive		33		32		30		32
Moderately active		45		46		49		49
Active		8		8		8		8
Education								
None		6		7		4		5
Primary school		41		42		41		44
Technical/professional school		23		24		26		24
Secondary school		14		12		14		11
Longer (including University)		16		15		16		15

Abbreviations: BMI, body mass index; OHA, oral hypoglycemic agent.

a Continuous variables are shown as mean (SE) and categorical variables are shown as %.

Diabetes medications such as insulin and oral hypoglycemic agents (OHA) are for most individuals with diabetes the main therapeutic option for glycemic control [1]. Glycemic regulation is often easy to achieve in the first few months after diagnosis, but becomes more difficult as the disease progresses [14]. Both the ADA and International Diabetes Federation (IDF) recommend OHA when lifestyle interventions are unable to maintain glycemic control in type 2 diabetes patients. Metformin is the drug of first choice, but when insufficient, therapy should be augmented with additional agents from different classes, and finally insulin therapy. In type 1 diabetes patients, insulin therapy is the only treatment option [1], [15]. Thus, different classes of medication appear to be effective at different stages of the disease [16] and type of medication used will reflect the disease stage.

**Table 2:** Baseline Characteristics a of 4,516 Individuals with Diabetes Mellitus from the European Prospective Investigation into Cancer and Nutrition by Type of Medication Use.

Diabetes medication	%	Age (y)	Mean difference HbA_1c_ (%)^b^	Disease duration (y)	BMI (kg/m^2^)	Co-morbidities (%)
None	31	56.8 (6.6)	−0.7	5.8 (6.1)	29.2 (4.7)	12
Metformin (monotherapy)	5	57.9 (6.8)	0	6.5 (6.0)	30.4 (4.8)	12
Metformin (combined)	11	58.6 (7.1)	+0.8	9.1 (7.2)	29.6 (4.6)	14
Sulfonamides	19	59.4 (6.9)	+0.6	8.3 (6.6)	28.7 (4.5)	18
Combinations or other OHA	7	57.8 (6.3)	+0.3	7.6 (7.0)	29.8 (5.4)	15
Insulin and OHA combined	7	58.1 (6.0)	+0.6	10.7 (8.7)	29.8 (4.9)	14
Insulin therapy	19	55.9 (8.3)	+0.9	14.7 (10.8)	27.2 (4.9)	18

Abbreviations: BMI, body mass index; OHA, oral hypoglycemic agent.

a Means (SE) or percentages are shown;

b Mean differences in HbA_1c_ values are given compared with metformin monotherapy.

The objective of this observational study was to investigate the association between HbA_1c_ measured in stored blood samples and mortality in individuals with diabetes mellitus. Secondly, it was explored whether the association was influenced by medication use and disease duration or whether these factors were independent mortality risk factors.

**Table 3:** Hazard Ratios (95% CI) of Associations between HbA_1c_, Diabetes Medication use, Disease Duration and Total Mortality in Individuals with Diabetes.

	Cases	PY	HR a	95% CI	HR b	95% CI
**HbA_1c_ (** ***n*** **=4,345)**						
Q1	96	11,075	1	Referent	1	Referent
Q2	100	9,761	1.26	0.93, 1.70	1.16	0.85, 1.58
Q3	114	9,802	1.15	0.86, 1.54	1.03	0.77, 1.39
Q4	150	9,495	2.02	1.53, 2.65	1.77	1.32, 2.36
P-Trend			<0001		<0001	
per 1% increase	460	40,133	1.13	1.08, 1.19	1.11	1.06, 1.17
**Diabetes medication (** ***n*** **=4,516)**						
No medication use	101	12,569	0.53	0.34, 0.81	0.58	0.37, 0.90
Metformin (monotherapy)	30	2,043	1	Referent	1	Referent
Metformin (combined)	68	4,653	0.72	0.46, 1.14	0.65	0.41, 1.04
Sulfonamides	153	7,545	0.82	0.54, 1.24	0.78	0.51, 1.18
Combinations or other OHA	30	2,573	0.76	0.44, 1.30	0.74	0.43, 1.27
Insulin and OHA combined	32	2,692	0.91	0.54, 1.56	0.79	0.46, 1.36
Insulin therapy	130	7,971	0.92	0.60, 1.41	0.76	0.49, 1.04
**Disease duration (** ***n*** **=5,837)**						
<2.0 y	157	13,695	1	Referent	1	Referent
2.0 – 4.6 y	170	13,567	1.01	0.81, 1.27	0.96	0.76, 1.21
4.6 – 9.9 y	191	13,212	1.27	1.02, 1.69	1.06	0.84, 1.34
>9.9 y	190	12,936	1.41	1.10, 1.82	1.08	0.83, 1.41
P-Trend			0.003		0.44	

Abbreviations: CI, confidence interval; HR, Hazard Ratio; OHA, Oral Hypoglycemic Agents; PY, person-years.

a Model 1: Age- and center-stratified and adjusted for sex, co-morbidities, physical activity, smoking status, educational attainment, body mass index, and systolic blood pressure;

b Model 2: Model 1 additionally adjusted for disease duration, diabetes medication use, or HbA_1c_ and storage time when adequate.

## Methods

### Study design

Within the European Prospective Investigation into Cancer and Nutrition (EPIC) [17], a sub-cohort was defined of participants with a confirmed diagnosis of diabetes mellitus at baseline. As has been described previously [18], [19], fifteen EPIC study centers from six European countries provided additional data on diabetes diagnosis and medication (Denmark, Germany, Italy, the Netherlands, Spain, and Sweden). Self-reports of diagnoses obtained at baseline were confirmed by additional information sources and included the following, dependent on the available option in study centers: contact to a medical practitioner, self-reported use of diabetes medication, repeated self-report during active follow-up (only in Potsdam, Germany), linkage to diabetes registries, or a baseline HbA_1c_ above 6% (measured in Malmö, Sweden only).

**Figure 2:**
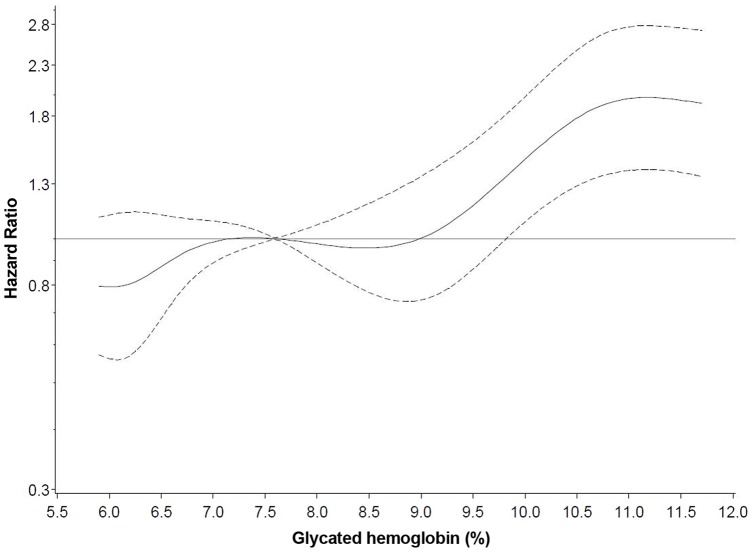
Adjusted Hazard Ratios of Death according to Glycated Hemoglobin (%) Measured in Stored Erythrocytes among 4,345 Individuals with Diabetes. Solid lines indicate hazard ratios and dashed lines indicate 95% confidence intervals derived from restricted cubic spline regression, with knots placed at the 5^th^, 10^th^, 25^th^, 75^th^, 90^th^, and 95^th^ percentiles of the distribution, using the 50^th^ percentile as a reference. Age- and study center-stratified models were adjusted for sex, storage time, disease duration, diabetes medication use, co-morbidities, physical activity, smoking status, educational attainment, body mass index, and systolic blood pressure. P value for nonlinearity derived from a Wald Chi-square test was P=0.15.

### Ethics statement

This study was conducted according to the guidelines laid down in the Declaration of Helsinki and was approved by ethical review boards of the single centres and the International Agency for Research on Cancer in Lyon, France. All subjects provided written informed consent.

**Table 4:** Hazard Ratios (95% CI) of Associations Between a 1% -Increase in HbA_1c_ and Total Mortality, Stratified for Several Diabetes-Related Variables, and Cause-Specific Mortality in 4,345 Individuals with Diabetes Mellitus.

	HR a	95% CI
per 1% increase	1.11	1.06, 1.17
**By diabetes medication**		
No medication	1.10	0.91, 1.34
OHA	1.13	1.05, 1.21
- Metformin	1.15	1.01, 1.31
- Sulphonamides	1.10	0.99, 1.22
Insulin and OHA	1.64	0.91, 2.94
Insulin therapy	1.14	0.98, 1.32
**By disease duration**		
<3.5 y	1.17	1.02, 1.33
3.5 – 9.9 y	1.09	1.00, 1.20
>9.9 y	1.08	0.99, 1.19
**By co-morbidities**		
With co-morbidities (11%)	1.09	0.96, 1.23
Without co-morbidities (89%)	1.11	1.04, 1.17
**Cause-specific mortality**		
CVD	1.14	1.05, 1.24
Cancer	1.05	0.95, 1.17
Other known causes	1.21	1.09, 1.35^b^

Abbreviations: CI, confidence interval; CVD, cardiovascular diseases; OHA, Oral Hypoglycemic Agents; HR, Hazard Ratio.

a Age- and center-stratified and adjusted for sex, physical activity, smoking status, educational attainment, body mass index, systolic blood pressure and for diabetes medication use, co-morbidities or disease duration when these were not stratified for.

b *P* value 0.04 for difference in risk estimate derived from competing risk model versus cancer mortality.

### Study population

Of the initial 7,048 self-reports of diabetes mellitus in the participating EPIC centers, a total of 6,412 participants were confirmed to have had diabetes at baseline. Of those, 27 were excluded due to missing mortality data. HbA_1c_ was measured in 4,345 of the remaining 6,385 participants (excluding all participants from Denmark). Furthermore, information on diabetes medication use was available for 4,516 (excluding all participants from Spain and Denmark) and disease duration for 5,837 participants.

In our data, the missingness of these variables could be explained by study center, which means they are missing at random. The assumption for a complete-case analysis is that the observations are missing completely at random. Thus, performing statistical analyses only on participants with complete data for these variables would have led to biased results. Nevertheless, performing multiple imputation for missing observation is valid when observations are missing at random; therefore, we created three different datasets for each exposure variable and applied multiple imputation on the missing observations among the covariates [20], [21]. Thus, the analytical sample of the three exposure groups comprised *n*=4,345 participants for HbA_1c_, *n*=4,516 participants for medication use, and *n*=5,837 participants for disease duration.

### HbA_1c_ measurement

HbA_1c_ was measured in erythrocytes of blood which was drawn at baseline between 1991 and 1998 in 4,345 participants from the study centers of Germany, Italy, the Netherlands, Spain, and Sweden. Blood samples were stored at −80°C and a part of EPIC-NL and EPIC-Italy blood samples was stored at −196°C. For all centers except Potsdam (Germany), both HbA_1c_ and hemoglobin were measured on an auto-analyzer (LX20-Pro, Beckman-Coulter) in July 2010; inter-assay coefficient of variation was 5.8%. In EPIC-Potsdam, HbA_1c_ was measured with the automatic ADVIA 1650 analyzer (Siemens Medical Solutions, Erlangen, Germany) in November 2008. To account for the long-term storage and differences in time of measurement, statistical analyses were adjusted for storage time, HbA_1c_ was analyzed in center-specific quartiles, and models with HbA_1c_ as a continuous variable were stratified for study center. Values were only interpreted in relative context, i.e. by ranking. Units are expressed as percentages of hemoglobin using the National Glycohemoglobin Standardization Program (NGSP).

### Diabetes-related medication

Information on insulin therapy or use of OHA was either self-reported at the visit at the study center or obtained during medical verification. Medication use was classified according to the Anatomical Therapeutic Chemical (ATC) classification of the World Health Organization. This information was not available for the study centers in Spain and Denmark. In addition, the lifestyle questionnaire included a question on insulin therapy. When a participant did not report the use of diabetes medication during the visit at the study center or did not report insulin therapy in the questionnaire, we assumed the participant did not take diabetes medication.

### Disease duration

Duration since diabetes diagnosis was calculated by subtracting the self-reported age at diagnosis or, when available, the exact date of diagnosis supplied by the medical practitioner from the age at baseline examination. Participants who reported an older age at diabetes diagnosis than their respective age at recruitment were excluded from the analyses with disease duration as a main exposure (*n*=109). In the other analyses, their disease duration was set to missing.

### Other covariates

Further lifestyle- and health-related variables were collected using questionnaires which included questions on smoking history, educational level, physical activity, and medical history including prevalent heart disease, stroke, and cancer. Weight and height as well as blood pressure values were measured at the baseline examination.

### Outcome ascertainment

Causes and dates of deaths were ascertained using record linkages with local, regional, or central cancer registries, boards of health, or death indexes (Denmark, Italy, the Netherlands, Spain, and Sweden). Germany identified deceased participants with follow-up mailings and subsequent inquiries to municipality registries, regional health departments, physicians, or hospitals. Mortality data were coded according to the *International Classification of Diseases, Injuries and Causes of Death*, Tenth Revision (ICD-10). For the cause-specific analyses, deaths due to circulatory diseases (ICD-10 codes I00-I99), cancer (ICD-10 codes C00-D48), and all other known causes were grouped accordingly. Deaths where the specific cause of death was unavailable (*n*=78) were included in the overall mortality analyses, but excluded from the cause-specific analysis.

### Statistical analyses

All statistical analyses were performed with SAS, version 9.2, software (SAS Institute, Inc., Cary, North Carolina). Hazard Ratios and 95% confidence intervals for mortality were calculated using Cox proportional hazard models [22]. The proportional hazard assumption was tested for HbA_1c_ with a Kolmogorov-type supremum test and was not violated. Centre and age at enrolment in 1-year categories were entered as stratum variables to control for differences in questionnaire design, follow-up procedures, and other non-measured center effects. Participants were followed from study entry until death, emigration, withdrawal, or the end of follow-up period. Age was used as the primary time variable with entry time defined as the subject's age in years at recruitment and exit time defined as the subject's age in years at death or censoring (lost to follow-up or end of follow-up period). Using age as the underlying time-scale and additional stratification on age at entry will control for period and cohort effects [23], [24]. All reported *P* values are 2 sided.

HbA_1c_ was analyzed continuously and in center-specific quartiles, using the first as reference. Diabetes medication was analyzed in categories of no medication, metformin monotherapy, metformin combined with other OHA, sulfonamides, other OHA or combinations, insulin and OHA combined, and insulin therapy (all coded as yes/no). Since metformin (monotherapy) is the preferred medication for type 2 diabetes [1], [15], this was used as reference. Disease duration was analyzed in quartiles using the first as a reference. Hazard Ratios were adjusted for sex, co-morbidities, smoking status (never/former/current), education (five categories), physical activity (four categories), body mass index (kg per m^2^), and systolic blood pressure (mmHg) (Model 1). Co-morbidities at baseline were defined as self-reported heart disease, stroke, cancer, or a follow-up of less than two years, since individuals with a short follow-up period were likely to have been severely ill at baseline. Model 2 was additionally adjusted for disease duration (years), diabetes medication (categorized as outlined above), or HbA_1c_ (%) and storage time (days) when adequate. A *P* value for trend across quartiles of HbA_1c_ and disease duration was calculated using the median value within categories as continuous variable. Statistical interaction between HbA_1c_, disease duration, and medication use was tested with a log likelihood ratio test by adding a product term to the model. The correlation coefficients between disease duration, age at recruitment, and length of follow-up were calculated, since these are time variables and may therefore lead to multicollinearity when too strongly correlated.

For HbA_1c_, the Wald Chi-Square test was used to test for nonlinearity. Risk for specific causes of death were derived from competing risk models in which separate regression coefficients for different causes of death were compared using the Wald Chi-square test and CI were derived from robust estimates of the covariance matrix [25], [26]. A restricted cubic spline regression model for the association between HbA_1c_ and mortality was fitted to provide further insight into the shape of the observed association. Knots were placed at the 5^th^, 10^th^, 25^th^, 75^th^, 90^th^, and 95^th^ percentile, and the 50^th^ percentile was used as a reference [27]. To test for heterogeneity across countries a Q-test was performed and I^2^ was calculated.

As mentioned previously, there were some missing values in covariates: proportions of missing data for the HbA_1c_-analyses were 12% for medication, 12% for disease duration, 20% for systolic blood pressure, 7% for physical activity, 1% for educational attainment, and 2%, 7%, and 10% for self-reported history of heart disease, stroke, and cancer respectively. We assumed that these values were missing at random and imputed them with the multiple imputation technique [21]. All variables (exposure, outcome, and covariates) included in the Cox proportional hazard models were also included in the procedure. Ten duplicate datasets were sampled from their predictive distribution based on the observed data with the missing values replaced by imputed values. In a sensitivity analysis, the associations between HbA_1c_ and mortality were compared with estimates from a complete-case analysis (*n*=2,983) to investigate whether missing observations of the covariates influenced the effect estimates. The same was done for medication use and disease duration.

## Results

The 4,345 participants included in the HbA_1c_-analyses were followed for a median of 9.3 years. In these years, 460 participants died: 160 from CVD, 120 from cancer, 102 from other known and external causes and in 78 the cause of death was unknown.

**Table** **1** displays general characteristics of study participants across quartiles of HbA_1c_. Participants with higher HbA_1c_ had a longer disease duration compared with those with a lower HbA_1c_. Furthermore, participants with a high HbA_1c_ were more likely to use diabetes medications, in particular insulin and sulfonamides, have a higher BMI and systolic blood pressure and were more likely to have co-morbidities. **Table** **2** shows that participants receiving metformin (monotherapy) had shorter disease duration, higher BMI, and lower prevalence of comorbidities than participants using other medication types. Those who did not report medication use had the lowest HbA_1c_, shortest disease duration and the lowest prevalence of co-morbidities. In addition, participants who reported insulin therapy had the highest HbA_1c_, longest duration, lowest BMI, and highest proportion of co-morbidities. In **Figure** **1**, glycemic control, medication use, and presence of comorbidities are displayed across deciles of disease duration in years on the x-axis. Medication use, in particular insulin use, co-morbidities, and HbA_1c_ levels increase with longer disease duration.

**Table** **3** shows the HR for total mortality according to HbA_1c_, medication use and disease duration. High HbA_1c_ categories were associated with higher mortality: participants in the highest quartile had a HR of 2.02 (95% confidence interval (CI): 1.53, 2.65) compared with those in the lowest quartile. Additional adjustment for disease duration and medication use slightly attenuated the association. Furthermore, an increment of 1% across all ranges of HbA_1c_ was associated with a 11% higher mortality risk. Excluding those who did not report to use diabetes medication did not change the effect estimates: HR for a 1% increase was 1.13 (95% CI 1.07, 1.20). Compared with metformin monotherapy, other medications or combinations were not differentially associated with mortality. Those who did not report medication use had a lower mortality risk (Hazard Ratio (HR) 0.53, 95% CI: 0.34, 0.81), even after adjustment for HbA_1c_ and disease duration. Participants with longer disease duration had an increased mortality risk: those with duration longer than 9.9 years had a hazard ratio of 1.41 (95% CI: 1.10, 1.82). This association disappeared when additional adjusting for HbA_1c_ and medication. Correlation coefficients were *r*=0.09 between disease duration and age at recruitment and *r*=−0.08 for between duration and follow-up time.

A significant linear trend was observed with death rates when studying HbA_1c_ across quartiles and the dose-dependent association was further confirmed by the restricted cubic spline regression in **Figure** **2**. *P* for nonlinearity was 0.74 and mortality risk seemed to increase continuously with higher HbA_1c_ values. Furthermore, the higher mortality risk per 1%-increase in HbA_1c_ was found to be persistent across categories of medication use, disease duration, and co-morbidities (**Table** **4**). In addition, HbA_1c_ was statistically significantly associated with a 14% increased risk of CVD mortality and a 21% increased risk of dying from non-CVD/non-cancer causes. Most frequent causes of death included in latter category were diabetes mellitus and its complications, and diseases of the respiratory and digestive system.

No statistical interaction between HbA_1c_, disease duration, and HbA_1c_ and medication use was found; *P V*alues for interaction were *P*>0.05. No heterogeneity between countries was found for the associations between HbA_1c_ and mortality: I^2^ was 0% (95% CI: 0–67%) and Q=2.58 (*P*=0.063). Results from the complete-case analysis were comparable to those derived from the multiple imputation procedure. The hazard ratio for a 1%-increase in the third adjustment model was 1.08 (95% CI: 1.02, 1.15) in the complete-case analysis, indicating that missing observations for any of the covariates did not influence the effect estimates.

## Discussion

This prospective study showed that HbA_1c_ measured in stored erythrocytes was positively linearly associated with mortality in individuals with a confirmed diagnosis of diabetes. This association was independent of disease duration, medication use, and presence of co-morbidities. Disease duration and medication use were not independently from HbA_1c_ associated with mortality.

We have shown that the association between HbA_1c_ and mortality was positive and linear across all concentrations in a large diabetic sample. There was a significant linear trend across quartiles of HbA_1c_, the Wald Chi-Square test gave no indication for nonlinearity of the association, and from the restricted cubic spline regression no threshold for HbA_1c_ could be detected from which mortality risk was increased. Recent findings from a randomized controlled trial [10], [28] and an observational study [9] have suggested that the association between HbA_1c_ and mortality might be U-shaped, i.e. individuals with low as well as high HbA_1c_ values have an increased risk of death. Other observational studies have shown similar results in persons with diabetes [3], [5]–[8], [29], [30] and without diabetes [29]–[32]. Eeg-Olofsson *et* *al.* prospectively investigated HbA_1c_ as a risk factor for CVD and found that a higher HbA_1c_ was associated with increased CVD and total mortality, even with longer duration, previous CVD, and treatment with either OHA or insulin. Moreover, poor long-term glycemic control was associated with increased risk of dying from ischemic heart disease in newly diagnosed diabetes patients [5].

Several randomized controlled trials have investigated the effect of tight glycemic control (HbA_1c_ target ranging from 6.0–6.5%) over standard therapy (HbA_1c_ target 7.0–7.9%). Overall, intensive treatment was associated with reduced CVD incidence, in particular non-fatal myocardial infarction, but not with total and CVD mortality [4], [11]–[13]. Although it is not possible to directly compare achieved HbA_1c_ levels with HbA_1c_ levels observed in a cohort study, these trials have shown that when studying glycemic control, it is important to take into account individual disease characteristics, such as progression, presence of complications and co-morbidities [10].

Cause-specific analyses showed that a higher HbA_1c_ was associated with increased risk of mortality due to CVD as well as due to non-CVD/non-cancer causes. Because the latter category mainly included death due to diabetes mellitus or its complications, this would suggest that higher levels of HbA_1c_ are associated with micro- as well as macrovascular outcomes, which is in line with existing literature [3], [33].

To the best of our knowledge, this is one of the first observational studies to investigate disease duration as an independent risk factor for mortality. We have found that with longer disease duration, medication use and HbA_1c_ increased accordingly. However, disease duration was not associated with mortality after adjusting for glycemic control and medication use. Moreover, higher levels of HbA_1c_ were associated with increased mortality across all strata of diabetes duration. Thus, disease duration did not seem to be independently related to mortality in our study. Diabetes mellitus is a progressive disease and glycemic control worsens as it advances [14]. Therefore, combinations of several blood glucose lowering medications are often required to achieve glycemic control as patients progress through the natural history of the disease [16]. Risk of diabetes complications increases with longer disease duration [34] and with poorer glycemic control [35], but up till now it was unclear whether these two factors are independent. We found one other study which demonstrated that that HbA_1c_ was higher in subjects with longer duration of diabetes [36].

Our findings showed that type and proportion of medication use differed across disease stage, but medication use in itself did not seem a mortality risk factor. Paradoxically, compared with metformin therapy, no associations between medication types and mortality were observed when HbA_1c_ and disease duration were taken into account. These findings could have been biased by misclassification, since diabetes medication use was self-reported at baseline and information on transitions between treatment regimens during follow-up was not available. Furthermore, recommendations for diabetes medication depend on the individual's disease stage [1], [15]. As a result, in observational studies where participants are not randomized to a treatment and the type of medication used will reflect disease progression. Thus, confounding by indication occurs, i.e. differences in prognostic factors may exist between type of medication. The UKPDS has shown that metformin was less effective in controlling plasma glucose at six months, but the effect was more sustained at three years compared with sulfonylureas and insulin. Both insulin and sulfonylureas were shown to have an equal treatment effect over a six-year period, but metformin had a more favorable effect on CVD risk, which was possibly mediated through its effect on weight [14]. Furthermore, metformin has been associated with a lower risk of CVD, total mortality and cancer incidence compared with sulfonylureas and insulin [37], [38]. Currie *et* *al.* showed increased mortality risk for those taking insulin compared with OHA [9].

Our study benefits from the large sample size, the multi-center design and the verification of self-reported diabetes diagnoses. Although the study design could have led to heterogeneity due to differences in HbA_1c_ measurement and verification of diabetes diagnoses between countries, we did not detect any. Moreover, this was taken into account in the statistical analyses as much as possible by stratifying by center. Further limitations which should be taken into account in the interpretation of the results are that diabetes medication and disease duration were mostly self-reported; therefore, misclassification may have occurred. Second, no information was available on treatment adherence and whether the prescribed medication was the most appropriate for the individual patient. Since it is known that compliance to both OHA and insulin is poor in many diabetes patients [39], no conclusions about the efficacy of different medication types can be drawn. Third, HbA_1c_ was measured in blood samples which were stored up to 19 years. HbA_1c_ values measured in long-term stored blood samples have found to be highly reliable [40]–[42], but small systematic differences may occur due to hemolysis during storage [41], [42]. However, since measurements were highly reliable, ranking of people would be unchanged and the relative measures of association unbiased [41], [42]. It was not possible to assess the direction or magnitude of bias caused by measuring HbA_1c_ in stored erythrocytes rather than fresh blood samples in our study. Furthermore, no follow-up information on changes in HbA_1c_ was available; therefore, we could not evaluate changes in management which have likely occurred. However, this study comprises individuals with diabetes at different ages and different stages of disease progression, thus covering the diversity of the diabetes population. Fourth, unfortunately information on presence of microvascular and macrovascular complications and on type 1 and type 2 diabetes was not available.

In conclusion, this observational study showed that HbA_1c_ from stored erythrocytes is positively and linearly associated with mortality in individuals with diabetes mellitus, independent from disease duration, medication use and presence of co-morbidities. Any improvement of HbA_1c_ appears to be associated with reduced mortality risk, also when taking into account disease duration.
